# Insulin resistance determined by Homeostasis Model Assessment (HOMA) and associations with metabolic syndrome among Chinese children and teenagers

**DOI:** 10.1186/1758-5996-5-71

**Published:** 2013-11-15

**Authors:** Jinhua Yin, Ming Li, Lu Xu, Ying Wang, Hong Cheng, Xiaoyuan Zhao, Jie Mi

**Affiliations:** 1 Department of Endocrinology, Key Laboratory of Endocrinology, Ministry of Health, Peking Union Medical College Hospital, Chinese Academy of Medical Sciences and Peking Union Medical College (CAMS & PUMC), Beijing 100730, China; 2 Department of Epidemiology, Capital Institute of Pediatrics, Beijing 100020, China; 3 Department of Endocrinology, First Affiliated Hospital, Shanxi Medical University, Shanxi 030001, China

**Keywords:** Homeostasis model assessment, Insulin resistance, Metabolic syndrome, Children, Teenagers

## Abstract

**Objective:**

The aim of this study is to assess the association between the degree of insulin resistance and the different components of the metabolic syndrome among Chinese children and adolescents. Moreover, to determine the cut-off values for homeostasis model assessment of insulin resistance (HOMA-IR) at MS risk.

**Methods:**

3203 Chinese children aged 6 to 18 years were recruited. Anthropometric and biochemical parameters were measured. Metabolic syndrome (MS) was identified by a modified Adult Treatment Panel III (ATP III) definition. HOMA-IR index was calculated and the normal reference ranges were defined from the healthy participants. Receiver operating characteristic (ROC) analysis was used to find the optimal cutoff of HOMA-IR for diagnosis of MS.

**Results:**

With the increase of insulin resistance (quintile of HOMA-IR value), the ORs of suffering MS or its related components were significantly increased. Participants in the highest quintile of HOMA-IR were about 60 times more likely to be classified with metabolic syndrome than those in the lowest quintile group. Similarly, the mean values of insulin and HOMA-IR increased with the number of MS components. The present HOMA-IR cutoff point corresponding to the 95th percentile of our healthy reference children was 3.0 for whole participants, 2.6 for children in prepubertal stage and 3.2 in pubertal period, respectively. The optimal point for diagnosis of MS was 2.3 in total participants, 1.7 in prepubertal children and 2.6 in pubertal adolescents, respectively, by ROC curve, which yielded high sensitivity and moderate specificity for a screening test. According to HOMA-IR > 3.0, the prevalence of insulin resistance in obese or MS children were 44.3% and 61.6% respectively.

**Conclusions:**

Our data indicates insulin resistance is common among Chinese obese children and adolescents, and is strongly related to MS risk, therefore requiring consideration early in life. As a reliable measure of insulin resistance and assessment of MS risk, the optimal HOMA-IR cut-off points in this cohort were developed with variation regarding puberty. HOMA-IR may be useful for early evaluating insulin resistance in children and teenagers and could have a long-term benefit of preventive and diagnostic therapeutic intervention.

## Background

Childhood obesity has experienced an important increase all over the world. It has been associated with the rising prevalence of many metabolic complications, such as hyperlipidemia, hyperglycemia and high blood pressure [1]. Many of them are already present during childhood and tend to persist into adulthood or further develop into metabolic syndrome (MS), and therefore increase the risk for development of cardiovascular disease (CVD) [2].

Insulin resistance is the primary metabolic disorder associated with obesity and appears to be the primary mediator of MS [3]. Identification of children with insulin resistance has been proposed as a strategy for identifying high-risk children for targeting MS interventions. The gold-standard technique for assessment of insulin sensitivity is the hyperinsulin-emiceuglycemic clamp [4]; and another accepted method is the minimal-model analysis frequently sampled intravenous glucose tolerance test (FSIVGTT) [5]. These tests are invasive, labor intensive, and expensive, which can be used for research purposes only. As a more convenient method to measure insulin resistance, the homeostasis model assessment of insulin resistance (HOMA-IR) was therefore developed and widely used in clinical and epidemiologic studies [6,7].

In children and adolescents, HOMA-IR has been validated as a surrogate measure of insulin resistance in several studies, showing high correlations with clamp or FSIVGTT measures [8,9]. However, it is more difficult to define HOMA-IR cut-off points for diagnosis of insulin resistance in youths than in adults, because there is lack of longitudinal evidence in youths for risk prediction of cardiovascular outcomes. Alternatively, in most studies, cut-off points for diagnosis of insulin resistance have been defined based on HOMA-IR distribution in reference population, but due to the influence factors such as puberty development and ethnic difference, values varied obviously from 1.8, 2.5, 3.2, to >4 according to the different reference population [10-12]. On the other hand, presence of pediatric MS, as a risk for future CVD, has been considered alternatively for defining cut-off values of HOMA-IR [9], but population-based studies are limited and there even exists debates on how and to what extent IR is associated with MS and its components.

In this context, our study aims are to evaluate the association of IR with each of the components of MS and to determine HOMA-IR cut-off values of different pubertal status regarding the diagnosis of MS based on a large cohort of Chinese schoolchildren. To our knowledge, there is lack of this kind of study in population- based samples of Chinese children.

## Methods

### Subjects

The data obtained from a cross-sectional population based survey conducted in Beijing area (the BCAMS cohort study) were analyzed [13,14]. The BCAMS study evaluated the prevalence of obesity and related metabolic abnormalities (hypertension, hyperglycemia and dyslipidemia) from a representative sample of Beijing school-age children (n = 19593, ages 6–18 years, 50% boys) between April and October 2004. Within this large group, 4500 of them were identified as having one of the following disorders: overweight defined by body mass index (BMI), increased cholesterol (≥5.2 mmol/L), triglyceride (TG) ≥ 1.7 mmol/L or fasting glucose ≥5.6 mmol/L based on finger capillary blood tests. All of the high risk participants were recruited for the second time of medical examination. A parallel reference population of 1045 school-age children was also studied. A total of 3203 schoolchildren (1679 boys) who had completed the further examination without missing data on variables needed for defining the MS were included in the current study; among them 420 subjects were diagnosed with MS according to the modified criteria of Adult Treatment Panel III (ATP III) definition [13,15] and 1037 subjects with normal weight status and without any components of MS were included serving as reference population. Signed informed consent was obtained from participants and/or parents/guardians. The BCAMS study was approved by the Ethics Committee at the Capital Institute of Pediatrics in Beijing.

### Clinical and anthropometric measurements

Subjects’ height and weight were measured according to our standard protocol [16]. BMI was calculated as weight (kg) divided by height squared (m^2^). BMI was converted to age- and sex-standardized percentiles based on the Centers for Disease Control and Prevention 2000 growth charts, which are not race specific [17]. Subjects were classified as normal weight if BMI was 5th ~ 85th percentile, overweight if BMI was 85th and 95th percentile, or obese if BMI was above 95th percentile. Waist circumference (WC) was measured midway between the lowest rib and the top of the iliac crest. Measurements of right arm systolic and diastolic blood pressure (SBP and DBP) were performed 3 times 10 minutes apart and the mean values of the latter two measurements were recorded. Pubertal development was assessed by Tanner stage of breast development in girls and testicular volume in boys. A testicular volume equal to or greater than 4 ml in boys and onset of breast development in girls were accepted as the criteria for onset of puberty [18]. This assessment was performed visually by two pediatricians of the same gender as the child.

### Laboratory measurement

Venous blood samples were collected after an overnight (≥12 h) fast. The concentrations of plasma glucose (glucose oxidize method), triglycerides (TG), total cholesterol (TC), high-density lipoprotein cholesterol (HDL-C) and low-density lipoprotein cholesterol (LDL-C) were assayed using the Hitachi 7060 C automatic biochemistry analysis system. HDL-C and LDL-C were measured directly. Insulin was measured by monoclonal antibody based sandwich enzyme-linked immuno sorbent assay (ELISA) [19], developed in the Key Laboratory of Endocrinology, Peking Union Medical College Hospital, which had an inter-assay coefficients of variation (CV) of <9.0% and no cross-reactivity to proinsulin (<0.05%). Fat percentage (FAT%) was assessed by bioelectrical impedance analysis (BIA, TANITA TBF-300A).

### Definition of MS and its related components and calculation of HOMA-IR

The International Diabetes Federation (IDF) released the first definition of MS in children and adolescents in 2007. However, it positions central obesity as an essential element, this almost rules out the possibility of diagnosing MS in a normal weight individual. Therefore, a modified ATPIII definition was employed in our study, in which MS was defined by the presence of three or more of the following five components [13,15]: (1) central obesity defined as WC ≥ 90th percentile for age and gender (established based on the BCAM study); (2) elevated systolic and/or diastolic blood pressure ≥ 95th percentile for age, sex and height (according to the BCAMS study); (3) hypertriglyceridemia defined as TG ≥1.24 mmol/L, equal to the 90th percentile of the reference population; (4) low serum HDL-C (Low-HDL) defined as ≤1.03 mmol/L i.e., ≤ 5th percentile of the reference population and (5) impaired fasting glucose (IFG) defined as ≥ 5.6 mmol/L. Insulin resistance index was calculated by homeostasis model assessment of insulin resistance (HOMA-IR) as (fasting insulin mU/L) × (fasting glucose mmol/L) / 22.5.

### Statistical analysis

All statistical analyses were carried out using the Statistical Package for Social Sciences (SPSS version 15.0 for Windows, Chicago, IL, USA). All skewed distributions were log transformed for analysis. Results are expressed as mean ± standard deviation (SD). The mean values of variables studied by analysis of variance (ANOVA). Level of significance was accepted as *P* < 0.05.

Logistic regression generalized estimating equation models were used to predict the risk of metabolic syndrome and its components by quintile of HOMA-IR after adjustment for age, sex and pubertal stage.

Insulin resistance was defined based on a number of different thresholds, including HOMA-IR threshold above 95th percentile for healthy reference population and ROC analysis to find the cutoff of HOMA-IR among subjects with and without metabolic syndrome. ROC analysis is a formal method of assessment for considering trade-offs between sensitivity and specificity at various test cutoffs or thresholds. A test with perfect discrimination has a ROC plot that passes through the upper left corner (100% sensitivity and 100% specificity). The ROC plot for HOMA is closer to the upper left corner, indicating greater overall accuracy of the test. To determine the optimal thresholds, the point on the ROC curve with maximum Youden index [sensitivity-(1-specificity)], and the point with shortest distance value form the point (0, 1) [(1 - sensitivity)^2^ + (1 - specificity)^2^] were calculated [20]. These are the two most commonly used methods for establishing the optimal cut-off [21].

In order to provide more information for science research and clinic reference, HOMA-IR cut-offs of different pubertal stage from the 95th percentile for healthy reference population along with their corresponding sensitivity and specificity in ROC curves were also evaluated. The prevalence of insulin resistance stratified by weight status or metabolic syndrome components based on different thresholds was calculated by the χ^2^ test.

## Results

### Descriptive statistics

Anthropometric and metabolic parameters of the study population by number of MS components are presented in Table 1. This study included 3203 children and adolescents (1679 boys and 1524 girls) aged 6–18 years. The mean age’s ± SD of study population were 12.1 ± 3.0 years. More boys were metabolic disorder than girls(*P* < 0.05). As number of MS related components increased, mean fasting insulin and HOMA-IR, as well as BMI, WC, FAT%, SBP, DBP and TG were gradually significantly increased, while HDL-C was significantly decreased.

**Table 1:** Characteristics of the study population according to number of metabolic abnormalities

**Number of metabolic abnormalities**	**0**	**1**	**2**	**≥3**
N(M/F)	1340(663/677)	1106(630/476)	640(378/262)	420(229/191)
Age(ys)	11.71 ± 3.09^a^	11.96 ± 3.09^b^	11.80 ± 3.04^a^	12.30 ± 2.95^b^
Tanner stage	2.58 ± 1.37^a^	2.75 ± 1.44^b^	2.71 ± 1.49^b^	2.84 ± 1.44^c^
BMI (kg/m^2^)	18.72 ± 3.36^a^	21.72 ± 4.17^b^	25.11 ± 4.03^c^	27.66 ± 4.11^d^
WC (cm)	64.15 ± 8.74^a^	71.62 ± 11.18^b^	80.96 ± 10.92^c^	87.48 ± 10.97^d^
FAT%	19.28 ± 6.60^a^	24.64 ± 7.78^b^	29.64 ± 7.35^c^	31.79 ± 6.94^d^
SBP (mm Hg)	99.11 ± 10.73^a^	108.26 ± 11.98^b^	115.79 ± 12.47^c^	120.60 ± 11.54^d^
DBP (mm Hg)	62.13 ± 8.05^a^	68.46 ± 8.92^b^	72.77 ± 9.52^c^	76.25 ± 8.50^d^
TC (mmol/L)	4.06 ± 0.76^a^	4.09 ± 0.83^a^	4.09 ± 0.92^a^	4.21 ± 0.83^b^
TG (mmol/L)#	0.78 ± 0.21^a^	1.03 ± 0.51^b^	1.19 ± 0.56^c^	1.67 ± 0.80^d^
HDL-C (mmol/L)	1.55 ± 0.29^a^	1.41 ± 0.30^b^	1.28 ± 0.26^c^	1.10 ± 0.22^d^
LDL-C (mmol/L)	2.44 ± 0.70^a^	2.55 ± 0.75^a^	2.61 ± 0.84^b^	2.77 ± 0.73^c^
FBG (mmol/L)	4.91 ± 0.37^a^	5.13 ± 0.48^b^	5.18 ± 0.70^b^	6.41 ± 0.90^c^
Fasting Insulin (mU/L)#	6.47 ± 4.25^a^	10.11 ± 7.33^b^	13.20 ± 7.84^c^	20.04 ± 19.35^d^
HOMA-IR#	1.42 ± 0.96^a^	2.33 ± 1.77^b^	3.03 ± 1.81^c^	4.96 ± 5.48^d^

*Abbreviations*: BMI body mass index, *WC* waist circumference, *SBP* systolic blood pressure, *DBP* diastolic blood pressure, *TC* total cholesterol, *TG* triglycerides, *HDL-C* high-density lipoprotein cholesterol, *LDL-C* low-density lipoprotein cholesterol, *FBG* Fasting blood glucose, *HOMA-IR* homeostatic model assessment of insulin resistance. Data are expressed as mean ± SD. #Skewed distributions were logarithmically transformed for one-way ANOVA. Values in the same row with different superscript letters are significantly different, with P < 0.05, that is, difference groups with the same superscript letter in the same row are no statistical significant.

### Risk of suffering from metabolic disorders according to HOMA-IR quintiles values

Logistic regression models predicting the presence of the MS and its related components (metabolic disorders) by quintiles of HOMA-IR values after adjustments for sex, age, and pubertal stage are shown in Table 2. Using the group with HOMA-IR values below the 20th percentile as a reference, it is apparent that the “odds ratio (OR)” of developing MS or its related metabolic disorders increased according to a rise of insulin resistance. Participants in the highest HOMA-IR quintile group were about 60 times more likely to have MS than those in the lowest quintile group (*P*<0.001). The OR of WC, TG and fasting blood glucose increased rapidly with increasing HOMA-IR level.

**Table 2:** Odds ratios of suffering cardiometabolic risk factors according to HOMA-IR quintile in Chinese schoolchildren

**Cardiometabolic risk factors**	** HOMA-IR quintile (values)**				
	**Quintile1 (<0.94)**	**Quintile2 (0.95-1.59)**	**Quintile3 (1.60-2.31)**	**Quintile4 (2.32-3.44)**	**Quintile5 (>3.45)**
MS(ATP III)					
OR	Referent	3.53	10.69	21.92	57.06
95%CI	-	1.41-8.87	4.55-25.10	9.48-50.72	24.87-130.92
*P*	-	0.007	<0.001	<0.001	<0.001
WC ≥ 90th percentile					
OR	Referent	2.50	5.33	11.22	27.96
95%CI	-	1.81-3.47	3.90-7.30	8.20-15.37	20.20-38.69
*P*	-	<0.001	<0.001	<0.001	<0.001
HDL-C ≤ 5th percentile					
OR	Referent	1.33	1.91	2.88	4.01
95%CI	-	0.81-2.19	1.19-3.08	1.82-4.54	2.57-6.25
*P*	-	0.265	<0.001	<0.001	<0.001
TG ≥ 90th percentile					
OR	Referent	2.13	3.71	5.11	9.49
95%CI	-	1.50-3.03	2.65-5.19	3.66-7.15	6.82-13.22
*P*	-	<0.001	<0.001	<0.001	<0.001
FBG ≥ 5.6 mmol/L					
OR	Referent	1.78	3.65	4.69	9.83
95%CI	-	1.03-3.10	2.20-6.06	2.84-7.75	6.05-15.96
*P*	-	0.040	<0.001	<0.001	<0.001
BloodPressure ≥ 95th percentile					
OR	Referent	1.67	2.84	3.52	5.98
95%CI	-	1.15-2.42	2.00-4.03	2.48-4.98	4.25-8.41
*P*	-	0.007	<0.001	<0.001	<0.001

Values obtained through logistic regression, adjusted for gender, age and tanner stage.

### HOMA-IR threshold above 95th percentile for healthy reference population

The mean, standard deviation and HOMA-IR threshold above 95th percentile for a healthy reference population according to gender, age and pubertal status are given in Table 3. HOMA-IR threshold above 95th percentile for the total of healthy reference population (normal-metabolic subjects) is 3.0. We find no significant gender difference in HOMA-IR threshold above 95th percentile. In prepubertal stage, HOMA-IR cut-off value from 95th percentile is 2.6 and so are 3.2 in the pubertal period. Insulin sensitivity reduces in Tanner stage V and almost completely recovered by pubertal completion. The similar result is found in different age ranges. When age is below 10, most of children are in prepubertal stage, the HOMA-IR cut-off value from 95th percentile is 2.1. However, the HOMA-IR threshold above 95th percentile is rapidly increased to 3.2 when age is up 10 years and most children go into puberty.

**Table 3:** The 95th percentiles of HOMA-IR according to gender, age and tanner stage in normal weight children without related components of MS

	**N**	**Mean**	**Std. deviation**	**95th**
Total	1037	1.29	1.11	**3.0**
Sex				
Male	442	1.21	0.93	3.1
Female	595	1.34	0.83	2.9
Age(years)				
6-9	278	0.81	0.57	2.1
≥10	759	1.46	0.90	3.2
10-15	622	1.47	0.93	3.2
≥16	137	1.45	0.76	3.2
Tanner stage				
I	384	0.93	0.72	**2.6**
≥II	643	1.51	0.89	**3.2**
II-IV	518	1.54	0.93	3.3
V	125	1.37	0.66	2.7

Bold data: HOMA-IR threshold for insulin resistance defined by the method of 95th percentile of reference population (with or without stratification of pubertal status).

### The cutoff of HOMA-IR determined by ROC analysis among subjects with and without metabolic syndrome

**Figure 1:**
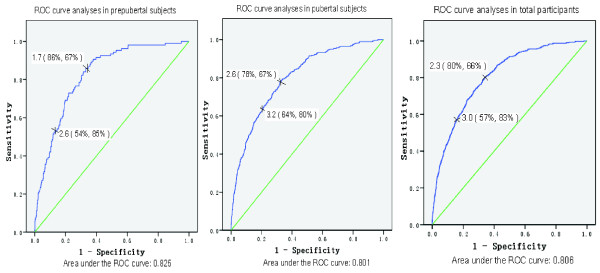
**ROC curve analyses of HOMA-IR cutoff in different Tanner stage children and adolescents. Left**: ROC curve analyses in prepubertal subjects. **Center**: ROC curve analyses in pubertal subjects. **Right**: ROC curve analyses in total participants.

In the total participants pool, the cut-off 2.3 was the best threshold for MS diagnosis by the modified ATP III definition. It maximized the Youden index and minimized the distance on the ROC curve (sensitivity = 80%, specificity = 66%, Youden index = 0.514, distance = 0.194). The area under the curves in the ROC analysis was 0.806. When considering puberty development, the cut-off 1.7 is the best threshold in the prepubertal period, (sensitivity = 86%, specificity = 67%, Youden index = 0.529, distance = 0.204). Furthermore, the pubertal period threshold is 2.6(sensitivity = 78%, specificity = 67%, Youden index = 0.458, distance = 0.153).Areas under the curves in the ROC analysis are 0.825 in prepubertal and 0.801 in pubertal period, respectively (Figure 1).

According to the cut-off points corresponding to the 95th percentile of HOMA-IR for normal subjects (3.0 for all, 2.6 for prepuberty and 3.2 for puberty, respectively), the sensitivity of those points for diagnosis of MS in ROC analysis are decreased 57%, 54% and 64%, respectively, while the specificity are increased to 83%, 85% and 80%, respectively.

### The prevalence of insulin resistance evaluated by different cutoffs of HOMA-IR

The prevalence of insulin resistance among those with ATPIII-defined MS and its related components according to our cutoffs using two different definitions in different pubertal period are given in Table 4. We find that, regardless of the definition used or the puberty status, the prevalence of insulin resistance was substantially higher in children with MS and its related components. For prepubertal children, using a threshold of insulin resistance of HOMA-IR > 1.7, the prevalence of insulin resistance in children with obesity or MS were 67.2% and 86.0% respectively; While using the threshold of HOMA-IR >2.6, the prevalence of insulin resistance were 39.0% and 53.3%, respectively. Similarly, for pubertal adolescents with obesity or MS, when HOMA-IR > 2.6, the prevalence of insulin resistance were 67.9% and 77.3%, and with a HOMA-IR > 3.4, the values were 46.3% and 59.2%, respectively.

**Table 4:** Prevalence of insulin resistance (%) according to various HOMA-IR cutoffs in cardio metabolic risk factors

		** Total(N** = **3203)**		** Prepuberty(N** = **1084)**		** Puberty(N** = **2119)**
**Cardiometabolic risk actors**	**2.3**	**3.0**	**1.7**	**2.6**	**2.6**	**3.2**
	**(ROC)**	**(>95th percentile in normal subjects)**	**(ROC)**	**(>95th percentile in normal subjects)**	**(ROC)**	**(>95th percentile in normal subjects)**
MS(ATP III)	79.0	61.6	86.0	53.3	77.3	63.5
WC ≥ 90th percentile	65.0	45.7	67.2	36.0	67.9	51.8
HDL-C ≤ 5th percentile	61.9	44.0	62.9	32.3	58.3	42.6
TG ≥ 90th percentile	59.9	42.5	67.7	38.9	55.1	42.2
FBG ≥ 5.6 mmol/L	65.2	47.7	62.0	34.0	64.5	51.6
BP ≥ 95th percentile	57.9	41.8	64.9	34.6	56.5	43.5

**Figure 2:**
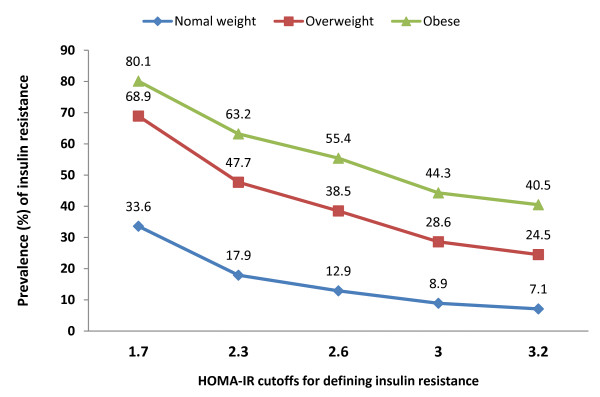
Prevalence of insulin resistance according to various HOMA-IR cutoffs in different weight status.

Figure 2 shows the prevalence of insulin resistance stratified by weight status based on different thresholds for defining insulin resistance. HOMA-IR 1.7, 2.6 or 2.3 were based on receiver-operator curve analysis with or without considering pubertal status. HOMA-IR 2.6, 3.2 or 3.0 were defined by above the 95th percentiles in normal-metabolic children and adolescents with or without considering puberty. Regardless of the definition used, the prevalence of insulin resistance was substantially higher in obese children compared with normal weight children. According to HOMA-IR 3.0 (95th percentile of our healthy reference), the prevalence of insulin resistance in obese adolescents was 44.3%.

## Discussion

The prevalence of obesity among children and adolescents is progressively increasing around the world. One of the important consequences of obesity is the development of insulin resistance (IR). Insulin resistance is a state in which normal concentrations of insulin produce a subnormal biologic response. This condition has a multifactorial pathogenesis and is associated with hyperlipidemia, hyperglycemia, high blood pressure and ovarian hyperandrogenism. Those are early state of adult diseases such as type 2 diabetes mellitus, hypertension, polycystic-ovary syndrome, cardiovascular disease and MS [22-24]. Although metabolic syndrome has been referred to as the insulin resistance syndrome, the ATPIII criteria do not include either fasting insulin level or the homeostasis model of insulin resistance (HOMA-IR) [15]. There has been debate about the extent to which the metabolic syndrome defines the risk of CVD associated with insulin resistance beyond the risk associated with classic CVD risk factors (obesity, HDL, triglycerides, and blood pressure) [25]. Therefore, it would be useful to understand the extent to which the presence of the syndrome is associated with insulin resistance.

In line with previous population-based studies [26,27], we found that insulin resistance and MS were significantly associated. HOMA-IR levels were directly related to the number of MS components and the risk of MS increased with rising HOMA-IR percentiles. As the number of MS related components increased, mean BMI, WC, SBP, DBP, FAT%, TG showed a gradually significant increase. Similarly, the mean insulin and HOMA-IR values increased with the number of MS components. From another point of view, we show the ORs of suffering MS according to IR categories. We use the group with HOMA-IR values below the 20th percentile as a reference, and we find that the odds of developing MS (adjusted for gender, age and tanner stage) increase as a function of IR. Participants in the highest quintile of HOMA-IR were about 60 times more likely to be classified with metabolic syndrome than those in the lowest quintile. Although the data are cross-sectional, it is not possible to identify the direction of causality among metabolic syndrome and HOMA-IR,and the relationship between insulin resistance and metabolic syndrome might be different in other samples. Nevertheless, the key implications are that youths with high insulin and HOMA-IR levels have a much greater risk of being classified with metabolic syndrome. Therefore, evaluation of insulin resistance as a pathological or physiological disease as well as early intervention will help control and reduce the currency of relevant diseases.

The correlation between HOMA-IR and M-clamp had been validated in diverse adult populations. Furthermore, two studies have described the pediatric information on its validation about clamps [8,9]. Although it is more difficult to define HOMA-IR cut-off points for diagnosis of insulin resistance in youths than that in adults due to lack of longitudinal evidence in youths for risk prediction of cardiovascular outcomes, there were a few studies on HOMA-IR utility in pediatric populations [28,29] and some methods for defining cutoff values of HOMA-IR. In most studies, cut-off points for diagnosis of insulin resistance have been defined based on HOMA-IR distribution in reference population. Values based on the 95th percentile [30-32], lower boundary of the top quintile [33,34] ortertile [35] of HOMA-IR obtained from population studies or non-obese subjects with no metabolic disorders have been used previously. In other studies, presence of pediatric MS, as a risk for future CVD, also has been considered for defining cut-off values of HOMA-IR by using ROC statistical method. Youden index and the distance from the top left corner of the ROC curve are two methods commonly used in previous work to determine the best HOMA-IR cut-off [33,36-38]. In our study, the present HOMA-IR cutoff point corresponding to the 95th percentile of our healthy reference children was 3.0 for whole referent and 2.6 for children in prepubertal stage and 3.2 in pubertal period, respectively. The optimal point for diagnosis of MS was 2.3 in total referent, 1.7 in prepubertal stage and 2.6 in pubertal period in ROC curve analysis.

Although HOMA-IR is wildly used in population-based studies, many factors involved in the inconsistencies of HOMA-IR should be stressed. Firstly, it is expected to be different in prepubertal and pubertal children as we show in this study. A transient insulin resistance develops in children during puberty. This insulin resistance emerging during pubertal maturation is accepted as a physiological condition rather than pathologic [39,40]. Some cross-sectional studies have shown that insulin resistance increases with the onset of puberty, makes a peak at Tanner stage 3 and recedes to prepubertal levels at the end of puberty [41-43]. Longitudinal studies have found a 30% decrease in insulin sensitivity between Tanner stages I and V [44]. However, this decrease was found to return to normal at the end of puberty [45]. In our study, while no difference for gender was detected in HOMA-IR cut-off values, it was higher in the pubertal period than that in the prepubertal period. A similar result was found in the different age phases; the HOMA-IR threshold is rapidly increased when children reach the age of 10 years, when most commence puberty. Therefore, it is important that, in the evaluation of insulin resistance in children and adolescents, different threshold values should be used according to puberty stage or age. Secondly, Different cut-off points might be selected to optimize sensitivity versus specificity depending on the study purpose. We defined a HOMA cutoff point for diagnosis of MS of 1.7 in prepubertal stage yielding a sensitivity of 86% and a specificity of 67% in the ROC curve. In pubertal population, the HOMA cutoff point of 2.6 produced a sensitivity of 78% and a specificity of 67%. A screening test requires high sensitivity and moderate specificity, whereas a diagnostic test requires a much higher specificity. In our study, the 95th percentile of HOMA-IR for normalsubjects in prepubertal stage was 2.6 and the sensitivity and the specificity of this point in the ROC analysis are were 54% and 85% respectively. The 95th percentile of HOMA-IR for pubertal adolescents of 3.2 leaded to a sensitivity of 64% and a specificity of 80%. Due to the fact that our sample size was large, we are able to propose precise cut-off limits based on the results of this study. This may be useful for different purposes, such as early intervention or early diagnose of insulin resistance in clinic. Thirdly, HOMA-IR is a function of both insulin and glucose, and glucose is included in the unified criteria of MS. However, insulin assays have not yet to be standardized and assessment methods differ between laboratories [46,47]. This makes comparison with different studies difficult.

## Conclusions

Our study determined HOMA-IR cut-off values of different pubertal status regarding the diagnosis of MS based on a large cohort of Chinese schoolchildren. Our results showed the prevalence of insulin resistance based on different thresholds stratified by weight status and cardio metabolic risk factors. The high prevalence of insulin resistance in obesity among Chinese children and teenagers, predicts an increasing burden of metabolic disease in the near future. HOMA-IR may be useful for early evaluating insulin resistance in children and teenagers and could have a long-term benefit of preventive and diagnostic therapeutic intervention [48].

### Limitations

Several limitations of our study should be acknowledged. Firstly, the results were limited by the use of HOMA rather than the gold standard technique, that is, hyperinsulin-emiceuglycemic clamp, for its complexity in large pediatric population. Secondly, there are numerous criticisms about the use of HOMA-IR, and the most important issue is the lack of standardization of insulin measurements, which makes comparison with other studies or population difficult. Thirdly, in our study, participants were children and adolescents predominately from Beijing, China. It is not possible to generalize the HOMA-IR cutoff to other ethnic populations.

## Abbreviations

HOMA-IR: Homeostasis model assessment of insulin resistance; MS: Metabolic Syndrome; ATP III: Adult Treatment Panel III; ROC analysis: Receiver operating characteristic analysis; FSIVGTT: Frequently sampled intravenous glucose tolerance test; CVD: Cardiovascular disease; IR: Insulin resistance; BMI: Body mass index; WC: Waist circumference; SBP: Systolic blood pressure; DBP: Diastolic blood pressure; TG: Triglycerides; TC: Total cholesterol; HDL-C: High-density lipoprotein cholesterol; LDL-C: Low-density lipoprotein cholesterol; IFG: Impaired fasting glucose; OR: Odds ratio.

## Competing interests

The authors declare that they have no competing interests.

## Authors’ contributions

JY carried out the study, data collection and analysis and drafted the manuscript. ML served as the major advisor and assisted in development, implementation and general advisement throughout the study. LX and YW made contribution to analysis and interpretation of data, HC and XZ attended to acquisition of data. JM served as co-advisor and conducted the collection of data. All authors read and approved the final manuscript.

## Authors’ information

JY is a PhD candidate with research focus on the area of obesity, metabolic syndrome and insulin resistance of children. LM is a professor and researcher focused on the research area of obesity, metabolic syndrome and insulin resistance in Department of endocrinology. LX is a PhD candidate and YW is a master candidate focused on obesity of children. HC and XZ are researchers in Department of Epidemiology of Pediatrics. JM is a professor and researcher in Department of Epidemiology.
